# Supplementary material for: The Interaction of Hydrogen with the van der Waals Crystal γ-InSe
Source: Molecules. 2020 May 28;25(11):2526. doi: 10.3390/molecules25112526 (PMC7321205; doi:10.3390/molecules25112526)
Supplement: Supplementary file 1 [file molecules-25-02526-s001.zip › supplementary material/Supplementary_Material.docx]

Version April 30, 2020 submitted to *Molecules*

S1 of S8

**Supplementary Materials: The interaction of hydrogen with the van der Waals Crystal** *γ***-InSe**

**James Felton 1,2,**∗**, Elena Blundo 3,Sanliang Ling2, Joseph Glover4, Zakhar R. Kudrynskyi1, Oleg Makarovsky1, Zakhar D. Kovalyuk5, Elena Besley4, Gavin Walker2, Antonio Polimeni3, Amalia Patané1,**∗

1

2

3

School of Physics and Astronomy, University of Nottingham, Nottingham NG7 2RD, UK Faculty of Engineering, University of Nottingham, Nottingham NG7 2RD, UK Dipartimento di Fisica, Sapienza Università di Roma, 00185 Roma, Italy

School of Chemistry, University of Nottingham, Nottingham NG7 2RD, UK

Institute for Problems of Materials Science, National Academy of Sciences of Ukraine, Chernivtsi Branch,

Chernivtsi 58001, Ukraine

e-mails: [james.felton1@nottingham.ac.uk,](mailto:james.felton1@nottingham.ac.uk) [amalia.patane@nottingham.ac.uk](mailto:amalia.patane@nottingham.ac.uk)

4

5

1

∗

2 **Abstract**

3 In the supplementary materials is shown: 1. The spot to spot variation of the low temperature PL

4 power dependence of pristine and hydrogenated *γ*-InSe. 2. The PL temperature dependence of pristine

5 and hydrogenated *γ*-InSe. 3. Thermodynamic simulations for incorporation of molecular hydrogen in

6 *γ*-InSe with different vdW gap spacings.

7


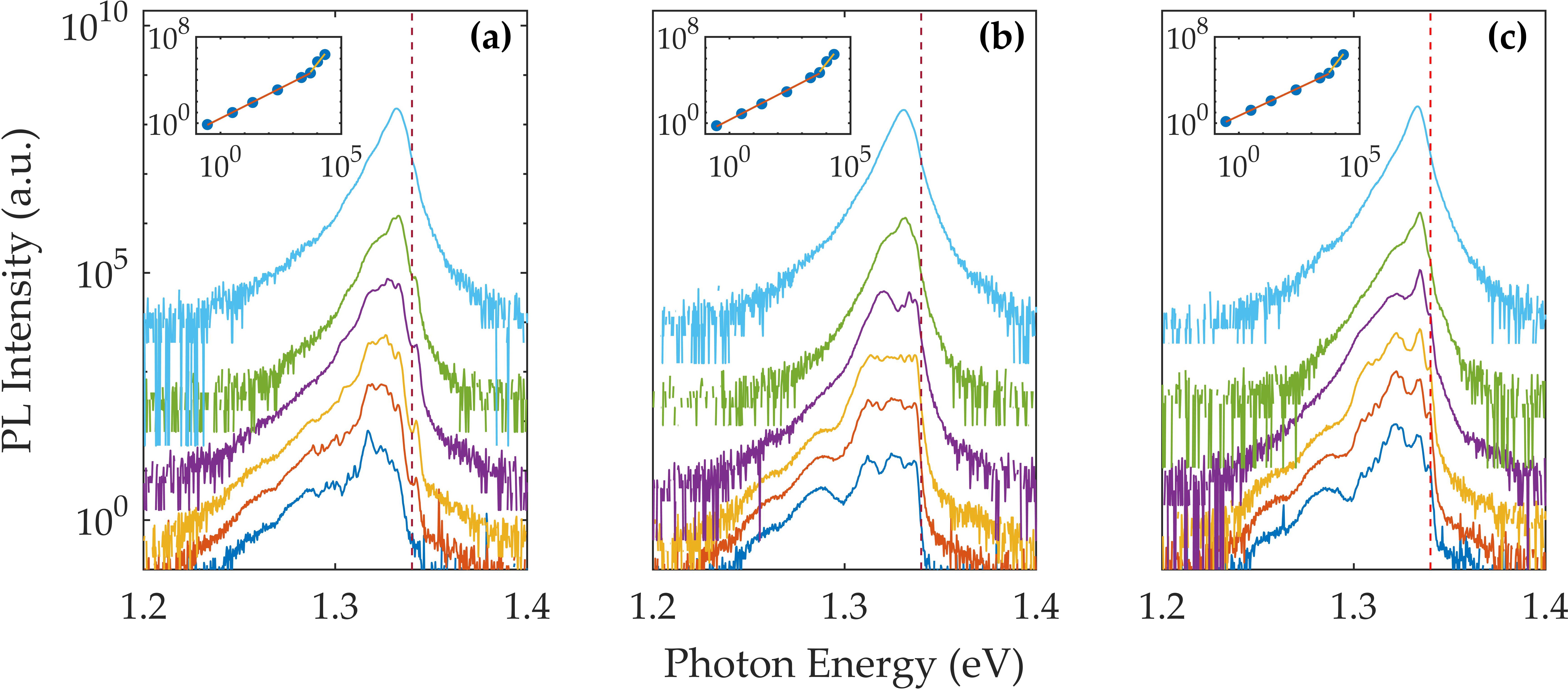

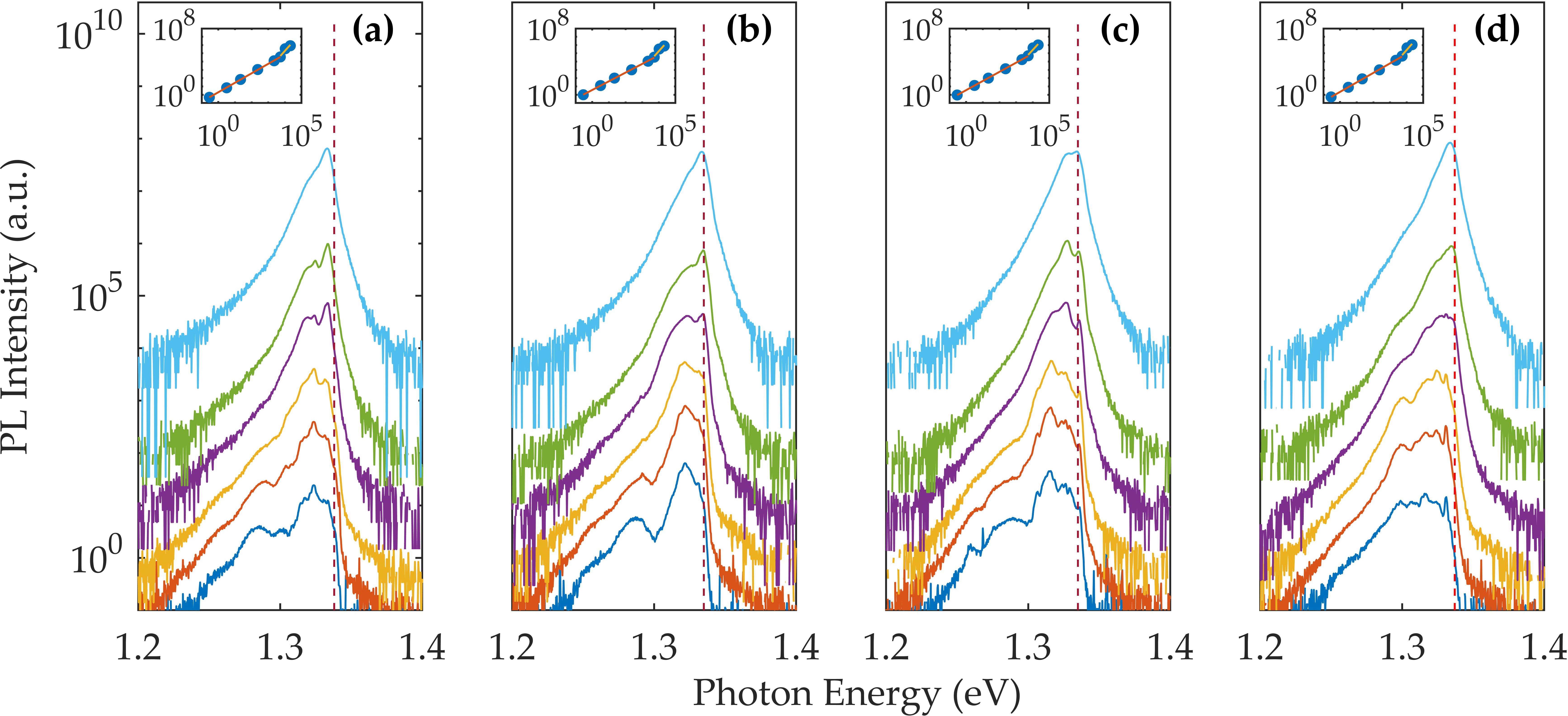


Version April 30, 2020 submitted to *Molecules*

S2 of S8

8 **1. Low temperature PL spot variation**

Low temperature PL was conducted on three spots in pristine *γ*-InSe and four spots in

9

10 hydrogenated *γ*-InSe, shown in Figures S1 and S2, respectively. The plots shown in Figures S1(c) and

11 S2(d) are the same as those in the main text. Comparing across different spots, the lack of distinct

12 hydrogen related features is apparent, as the variation within each sample is significant. These PL

13 spectra were measured using the methodology described in the main text.

**Figure S1.** PL measured in pristine *γ*-InSe at 10 K (*λ*=532 nm). Shown in (**a**), (**b**) and (**c**) are the spectra from different locations on the sample. Each spot has 6 spectra each gathered at a different power, the same as those in the main text (0.27 *µ*W, 2.9 *µ*W, 20.2 *µ*W, 0.22 mW, 2.05 mW and 19.5 mW). The dashed lines mark the proposed position of the exciton line. The insets show the total integrated intensity plotted against laser power in units of *µ*W.

**Figure S2.** PL measured in hydrogenated (10 eV) *γ*-InSe at 10 K (*λ*=532 nm). Shown in (**a**), (**b**), (**c**) and

(**d**) are the spectra from different locations on the sample. Each spot has 6 spectra each gathered at a different power, the same as those in the main text (0.27 *µ*W, 2.9 *µ*W, 20.2 *µ*W, 0.22 mW, 2.05 mW and

19.5 mW). The dashed lines mark the proposed position of the exciton line. The insets show the total integrated intensity plotted against laser power in units of *µ*W.


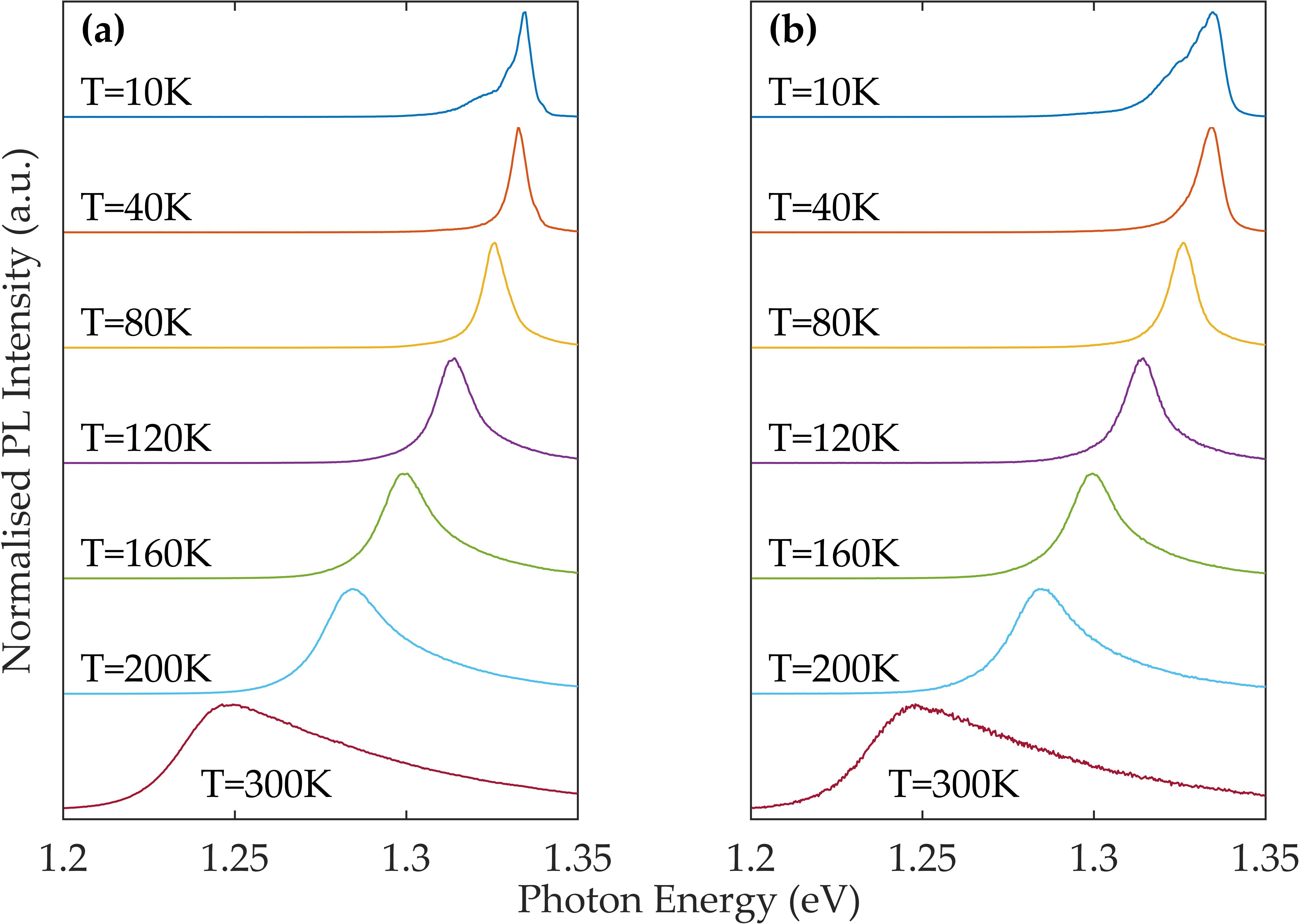


Version April 30, 2020 submitted to *Molecules*

S3 of S8

14 **2. Low temperature PL temperature dependence**

If molecular hydrogen is present in hydrogenated *γ*-InSe, then an associated phase transition

15

16 might be observed upon varying the temperature. Figures S3(a) and S3(b) show the temperature

17 dependence of the PL signals corresponding to the spots shown in Figures S1(c) and S2(d), respectively.

18 A small difference is apparent between the two samples at 10 K due to the reduced excitonic features

19 in hydrogenated *γ*-InSe. Otherwise, both samples PL signals progress in the same manner towards

20 their expected room temperature lineshapes. These PL spectra were measured using the methodology

21 described in the main text.

**Figure S3.** Normalised PL measured in (**a**) pristine and (**b**) hydrogenated (10 eV) *γ*-InSe at temperatures from 10 K to 300 K (*λ* = 532 nm, *P* = 2.05 mW).


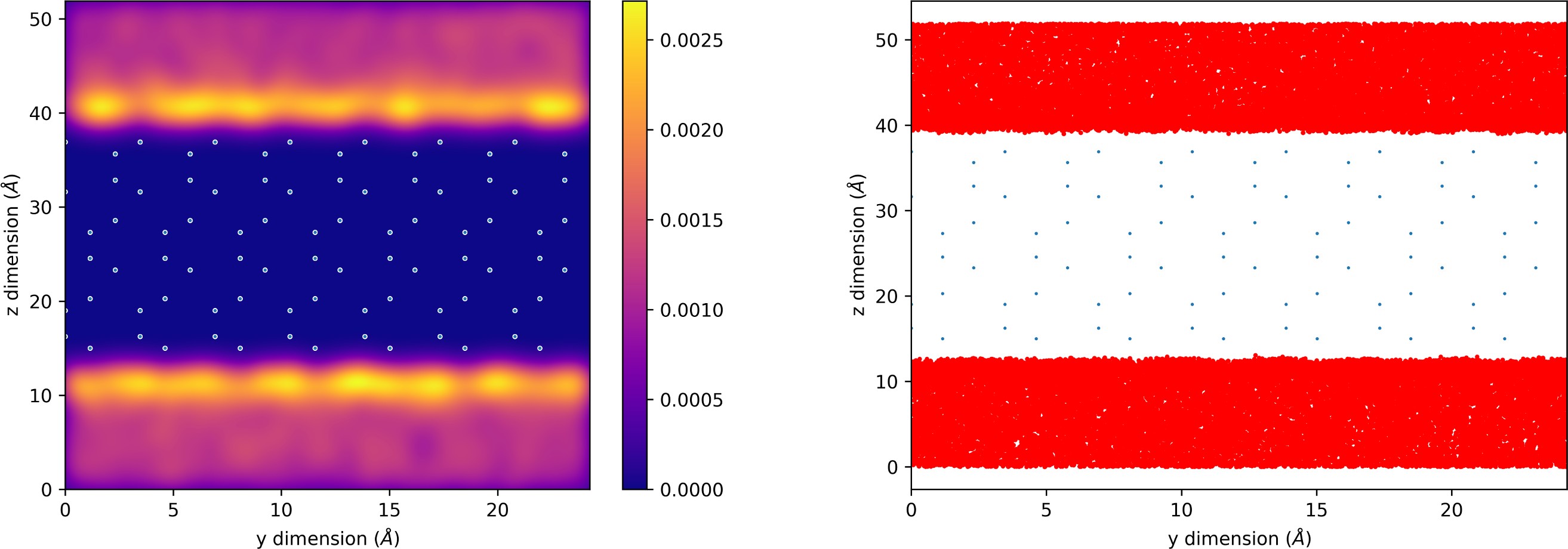

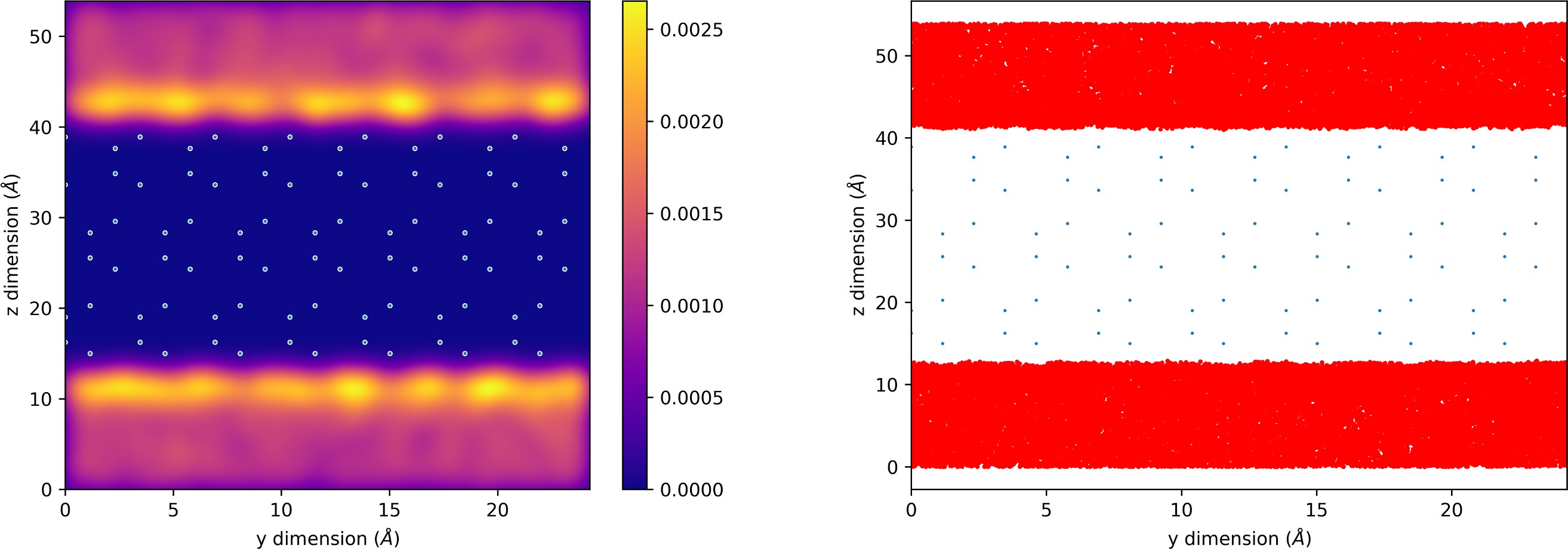


Version April 30, 2020 submitted to *Molecules*

S4 of S8

22 **3. Thermodynamic simulations**

Shown in Figures S4 to S13 are the simulated heatmaps and centre of mass coordinates for

23

24 molecular hydrogen incorporation in *γ*-InSe with increasing vdW gap sizes. Significant hydrogen

25 accumulation within the gap is not achieved until the gap is increased by 2.5 Å over pristine *γ*-InSe.

26 Shown in Figure S14 is the absorption isotherm for hydrogen in *γ*-InSe with differently sized vdW

27 gaps. A significant increase in density is seen for spacings above 2.5 Å. These results were aquired

28 using the methodology described in the main text.

**Figure S4.** In pristine *γ*-InSe: (**a**) H2 density heatmap created using kernel density estimation over 10,000 cycles in the adsorption isotherm calculations. (**b**) The H2 adsorbates centre of mass coordinates.

**Figure S5.** In *γ*-InSe with the vdW gap increased by 1 Å: (**a**) H2 density heatmap created using kernel density estimation over 10,000 cycles in the adsorption isotherm calculations. (**b**) The H2 adsorbates centre of mass coordinates.


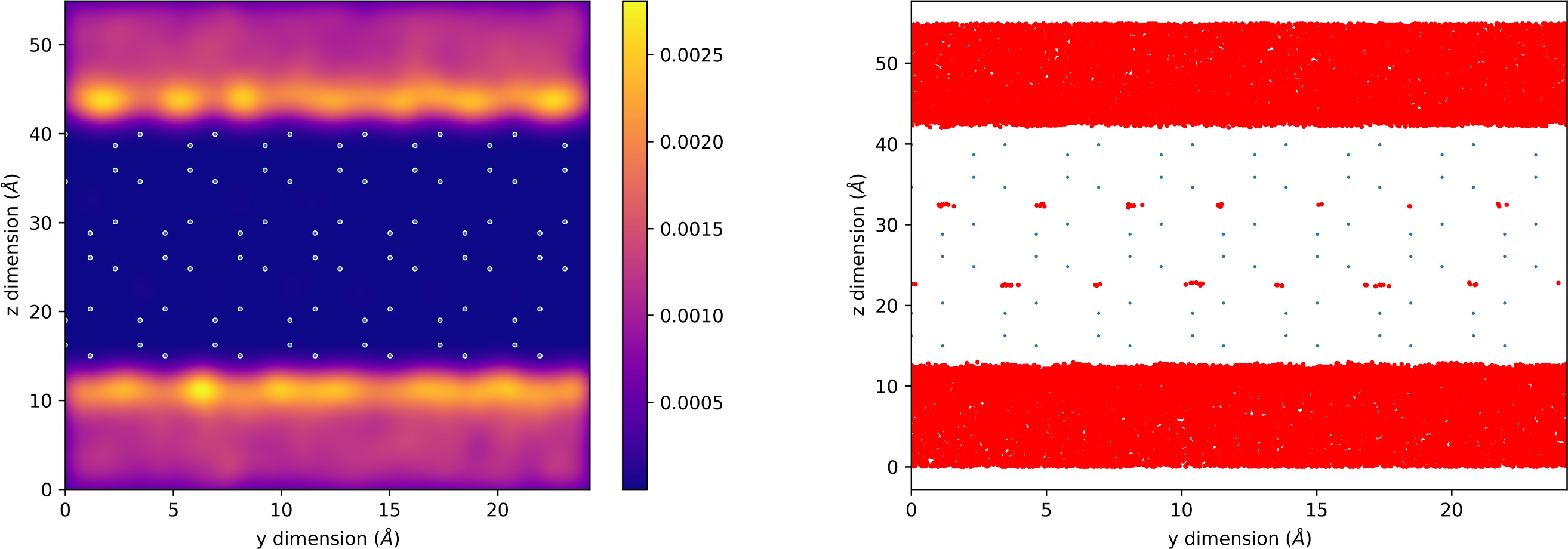

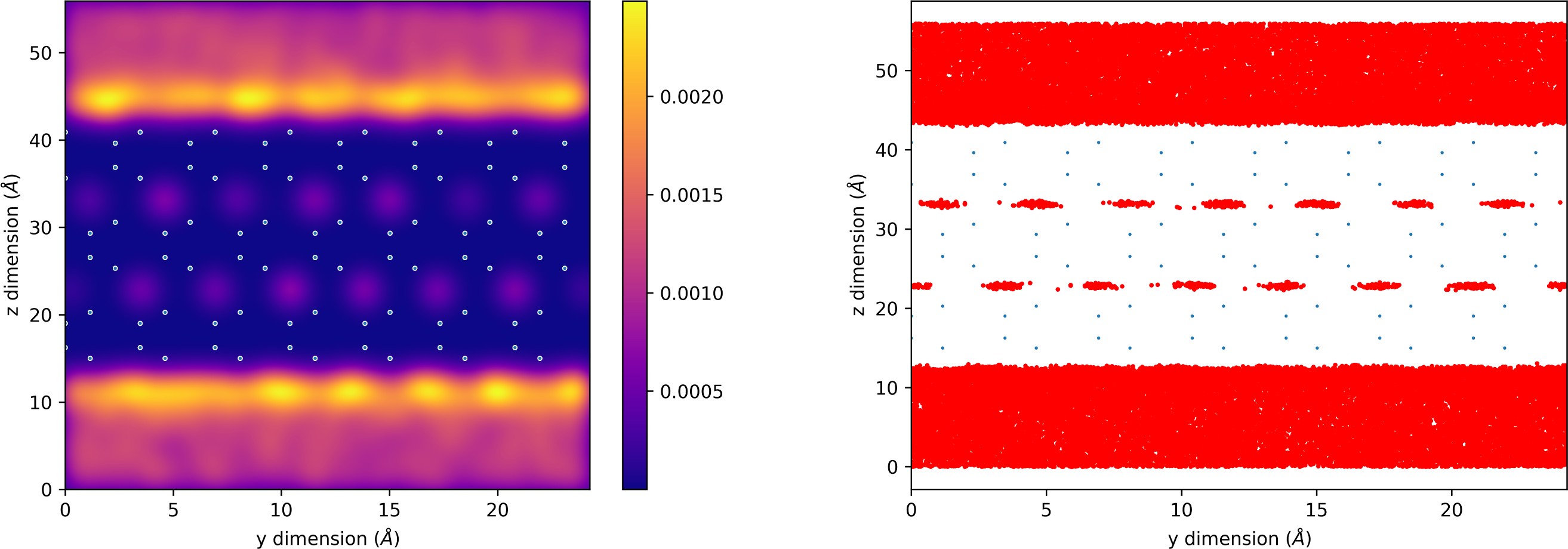

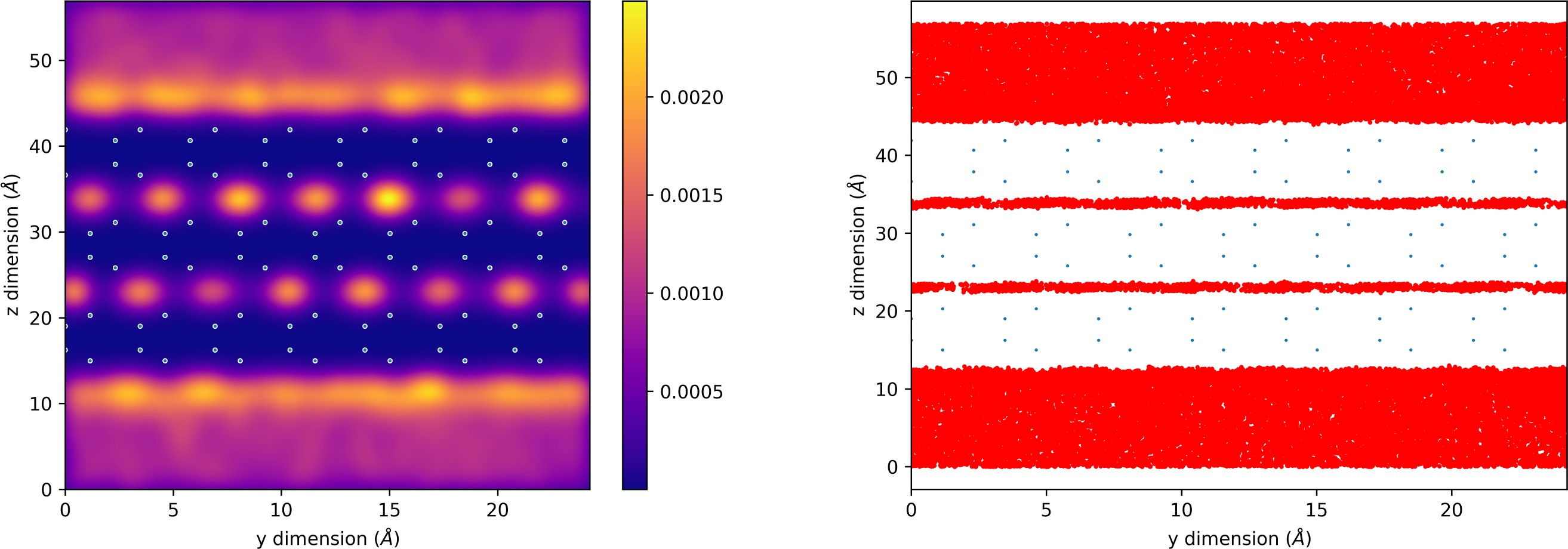


Version April 30, 2020 submitted to *Molecules*

S5 of S8

**Figure S6.** In *γ*-InSe with the vdW gap increased by 1.5 Å: (**a**) H2 density heatmap created using kernel density estimation over 10,000 cycles in the adsorption isotherm calculations. (**b**) The H2 adsorbates centre of mass coordinates.

**Figure S7.** In *γ*-InSe with the vdW gap increased by 2 Å: (**a**) H2 density heatmap created using kernel density estimation over 10,000 cycles in the adsorption isotherm calculations from. (**b**) The H2 adsorbates centre of mass coordinates.

**Figure S8.** In *γ*-InSe with the vdW gap increased by 2.5 Å: (**a**) H2 density heatmap created using kernel density estimation over 10,000 cycles in the adsorption isotherm calculations from. (**b**) The H2 adsorbates centre of mass coordinates.


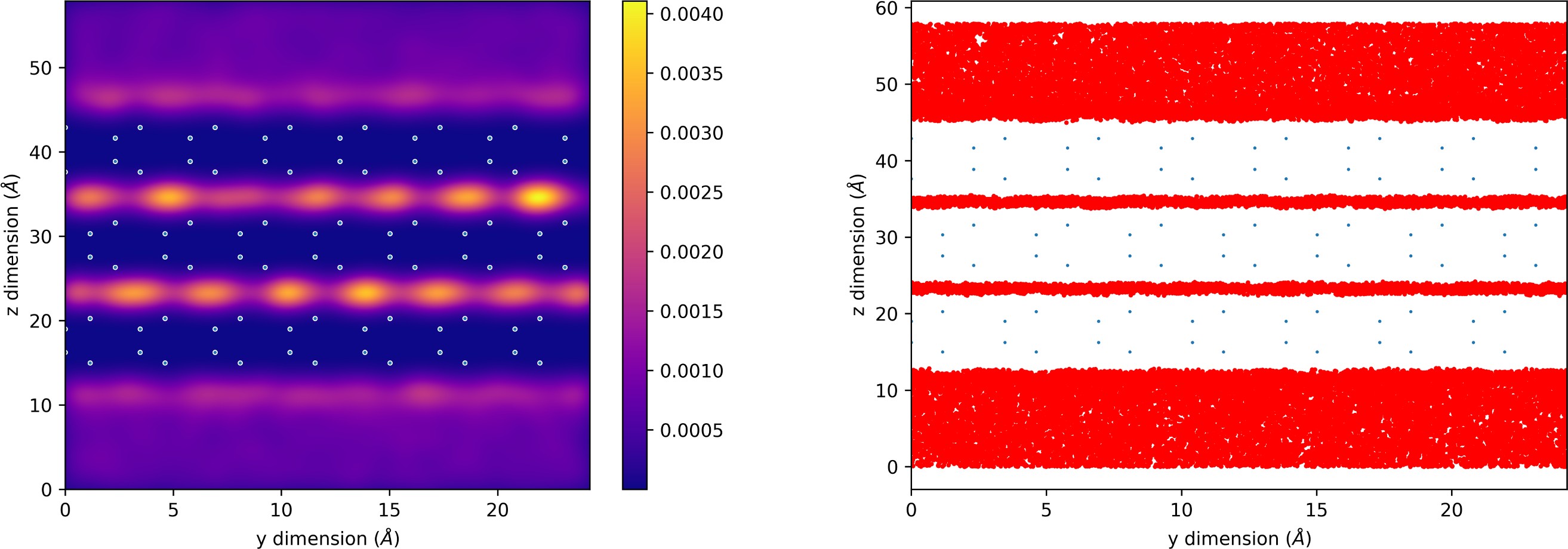

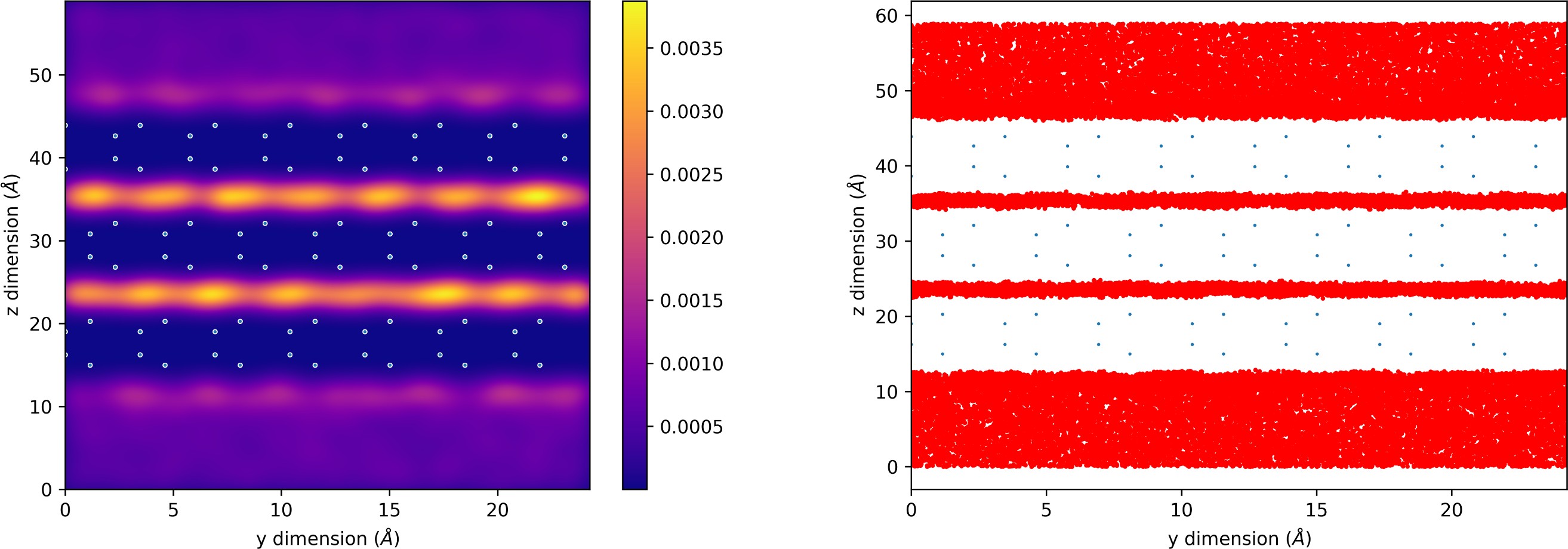

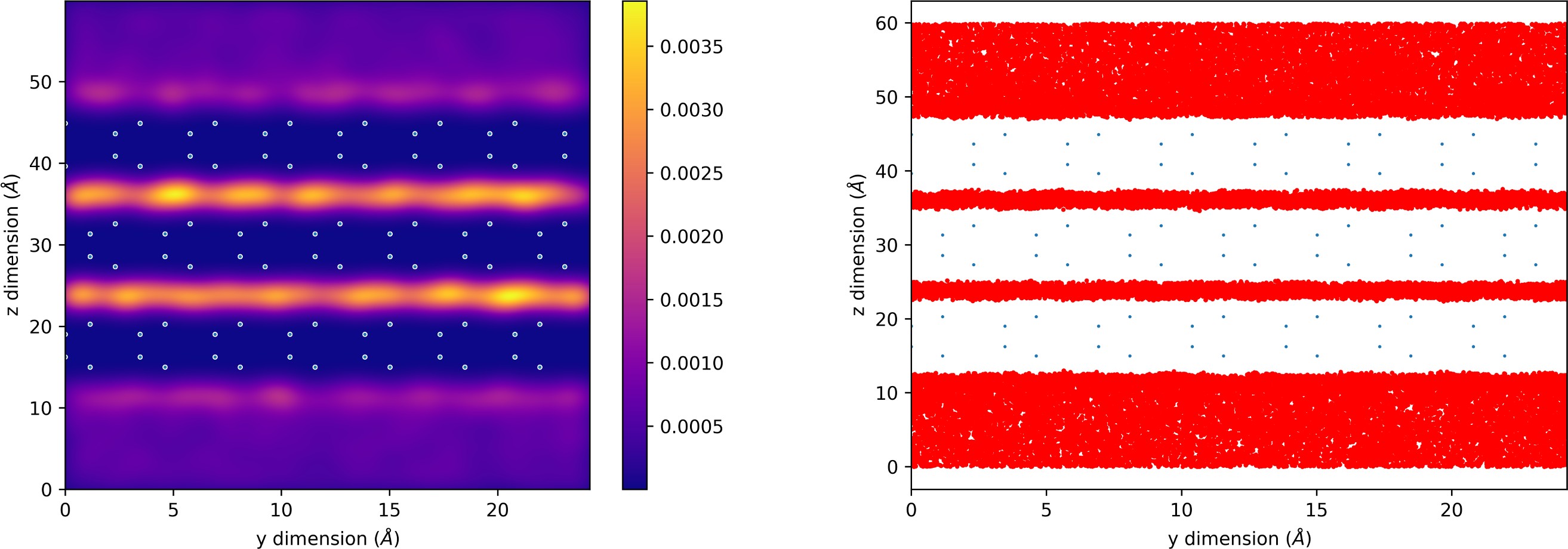


Version April 30, 2020 submitted to *Molecules*

S6 of S8

**Figure S9.** In *γ*-InSe with the vdW gap increased by 3 Å: (**a**) H2 density heatmap created using kernel density estimation over 10,000 cycles in the adsorption isotherm calculations from. (**b**) The H2 adsorbates centre of mass coordinates.

**Figure S10.** In *γ*-InSe with the vdW gap increased by 3.5 Å: (**a**) H2 density heatmap created using kernel density estimation over 10,000 cycles in the adsorption isotherm calculations from. (**b**) The H2 adsorbates centre of mass coordinates.

**Figure S11.** In *γ*-InSe with the vdW gap increased by 4 Å: (**a**) H2 density heatmap created using kernel density estimation over 10,000 cycles in the adsorption isotherm calculations from. (**b**) The H2 adsorbates centre of mass coordinates.


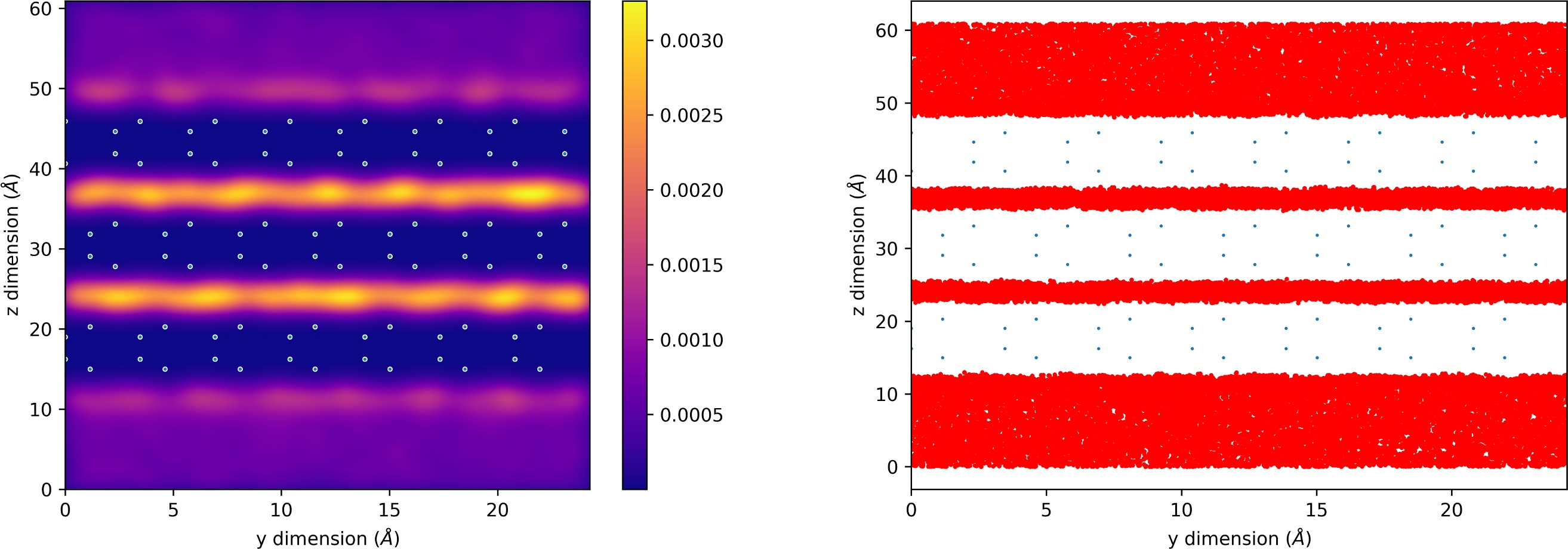

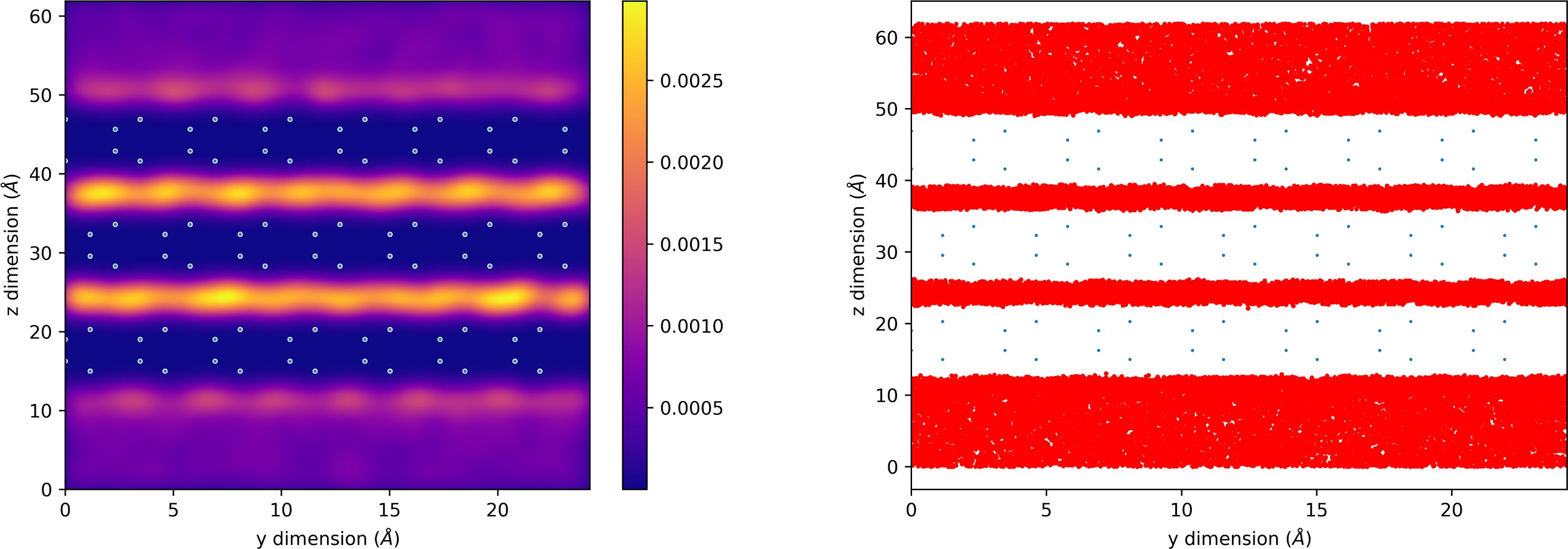


Version April 30, 2020 submitted to *Molecules*

S7 of S8

**Figure S12.** In *γ*-InSe with the vdW gap increased by 4.5 Å: (**a**) H2 density heatmap created using kernel density estimation over 10,000 cycles in the adsorption isotherm calculations from. (**b**) The H2 adsorbates centre of mass coordinates.

**Figure S13.** In *γ*-InSe with the vdW gap increased by 5 Å: (**a**) H2 density heatmap created using kernel density estimation over 10,000 cycles in the adsorption isotherm calculations from. (**b**) The H2 adsorbates centre of mass coordinates.


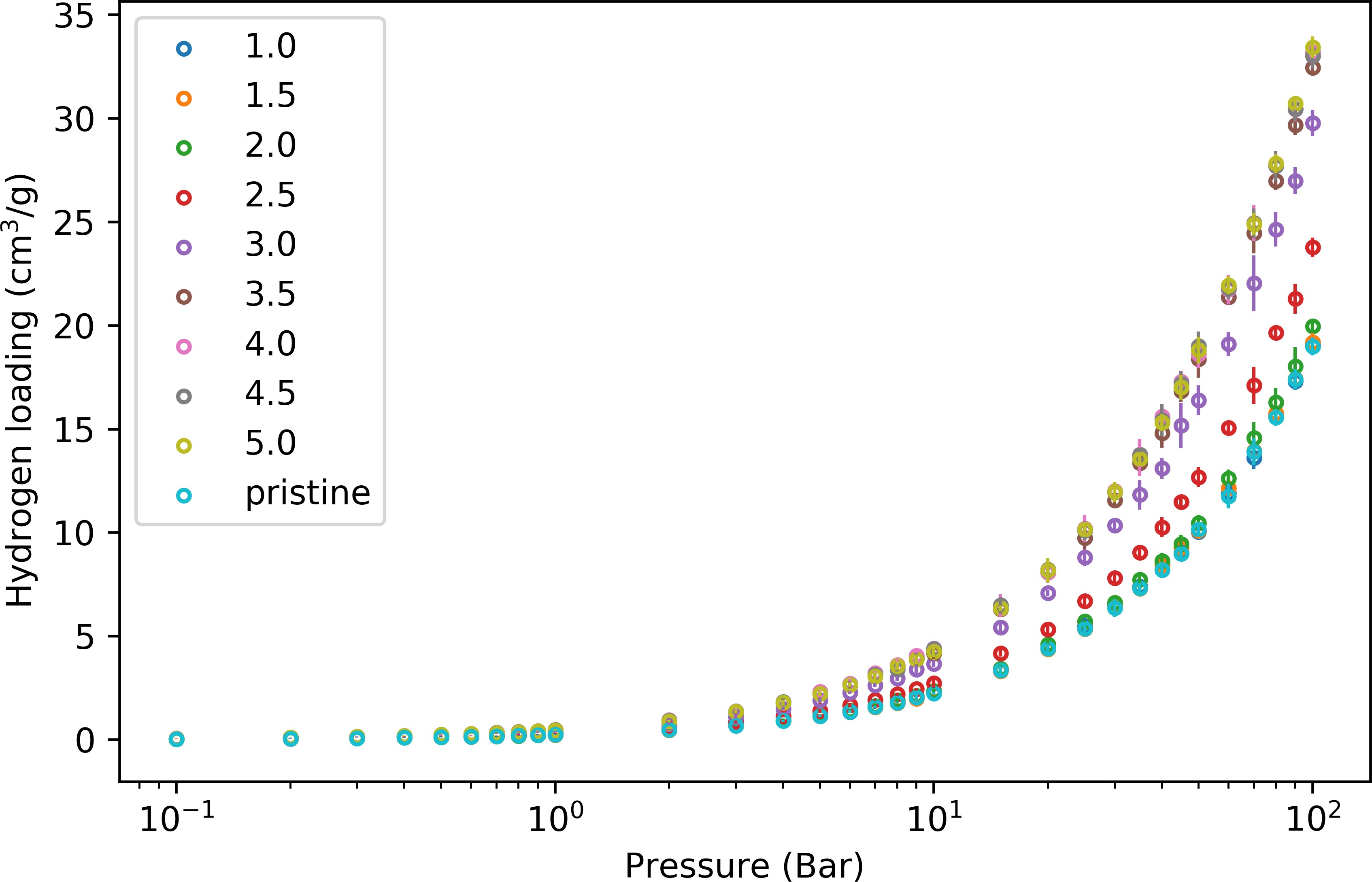


Version April 30, 2020 submitted to *Molecules*

S8 of S8

**Figure S14.** The hydrogen adsorption isotherm for *γ*-InSe with different vdW gap spacings. Each point is calculated using kernel density estimation over 10,000 cycles at 298 K. Interlayer spacings measured in Å.
