# Supplementary figures and images for: The Interaction of Hydrogen with the van der Waals Crystal γ-InSe
Source: Molecules. 2020 May 28;25(11):2526. doi: 10.3390/molecules25112526 (PMC7321205; doi:10.3390/molecules25112526)

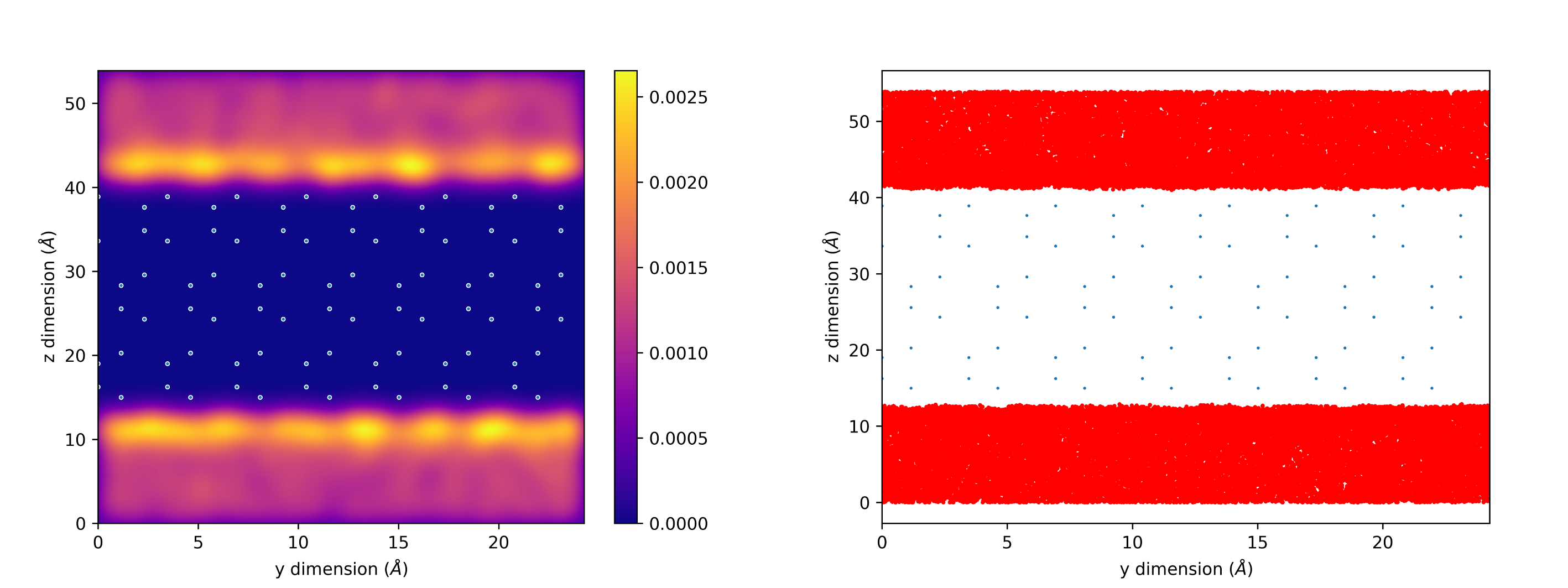

Supplement: Supplementary file 1 [file molecules-25-02526-s001.zip › supplementary material/1_0_joined.png]

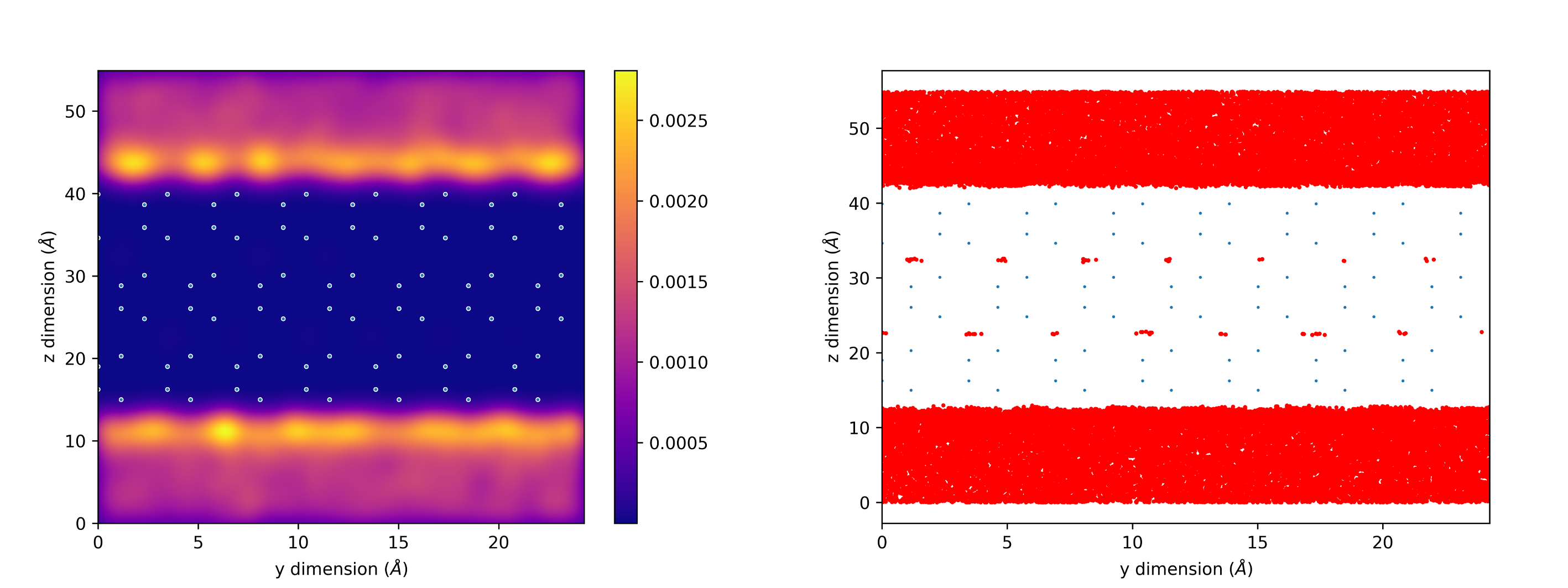

Supplement: Supplementary file 1 [file molecules-25-02526-s001.zip › supplementary material/1_5_joined.png]

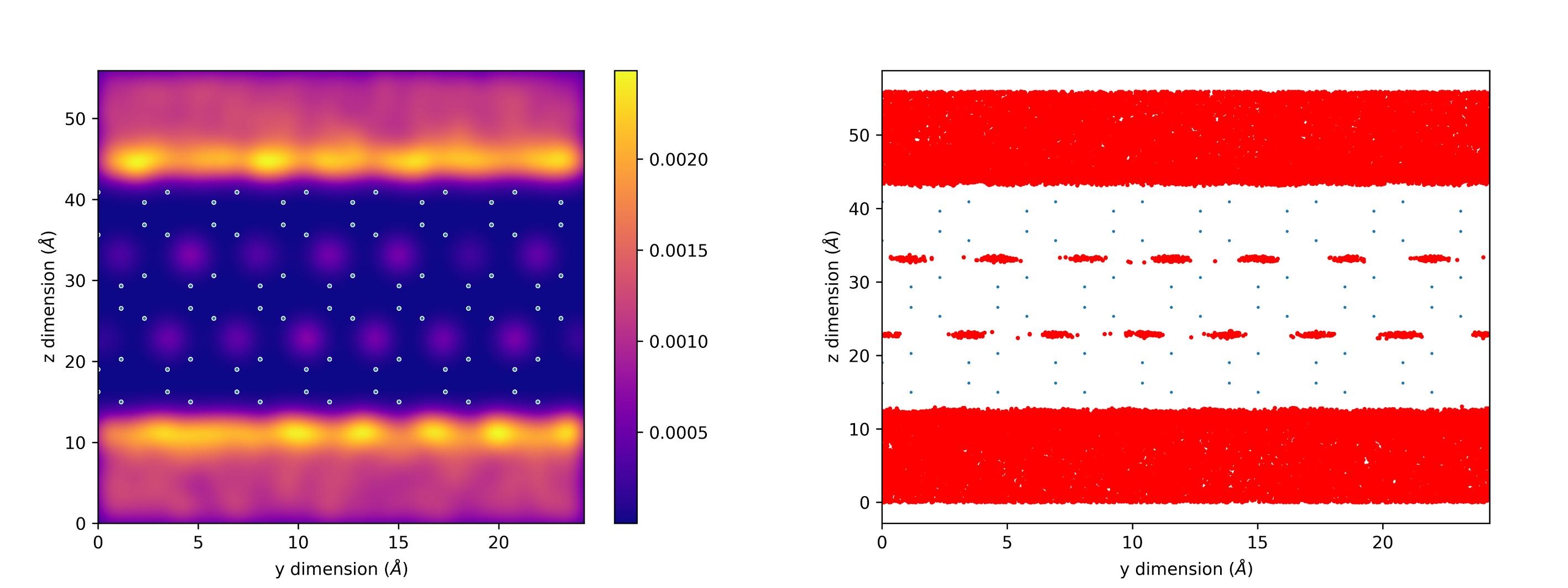

Supplement: Supplementary file 1 [file molecules-25-02526-s001.zip › supplementary material/2_0_joined.png]

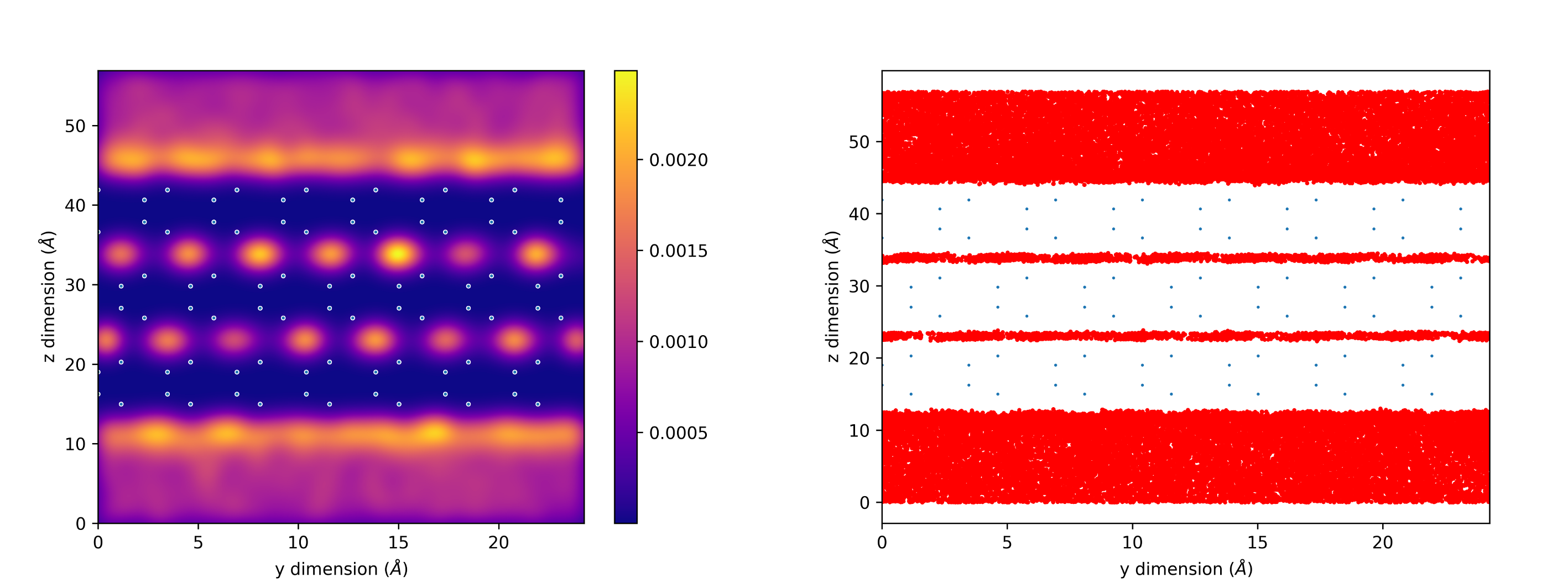

Supplement: Supplementary file 1 [file molecules-25-02526-s001.zip › supplementary material/2_5_joined.png]

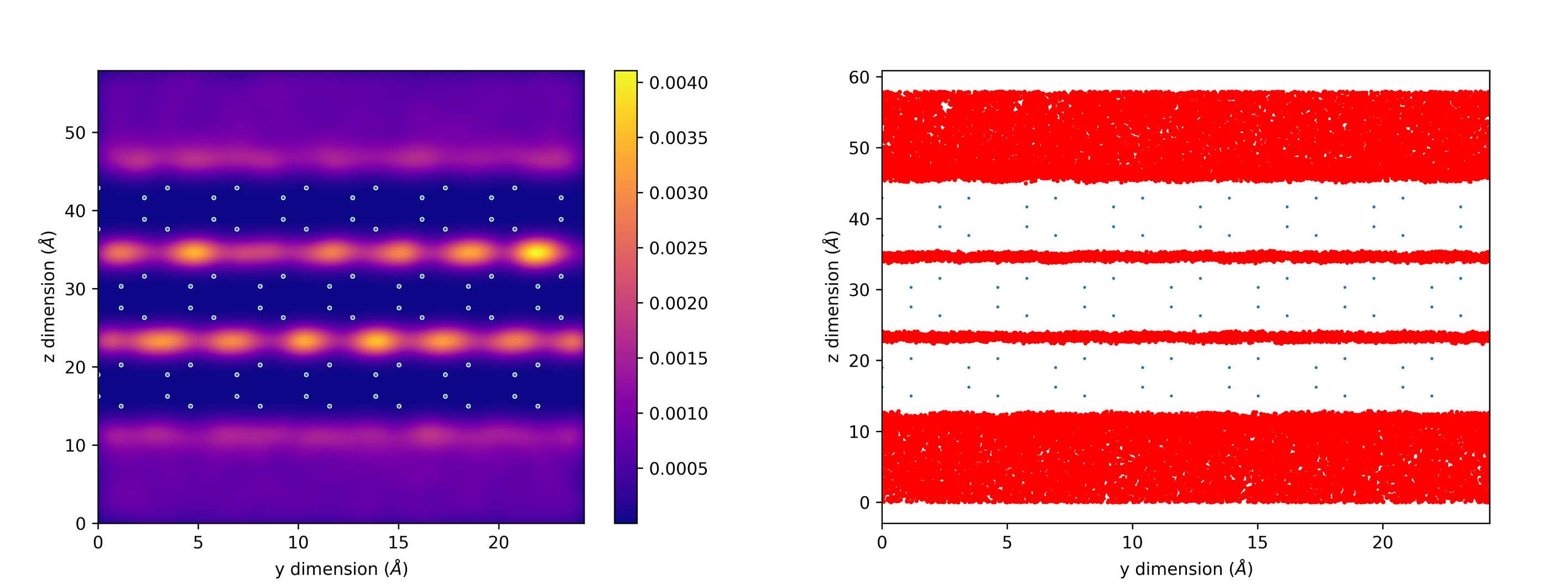

Supplement: Supplementary file 1 [file molecules-25-02526-s001.zip › supplementary material/3_0_joined.png]

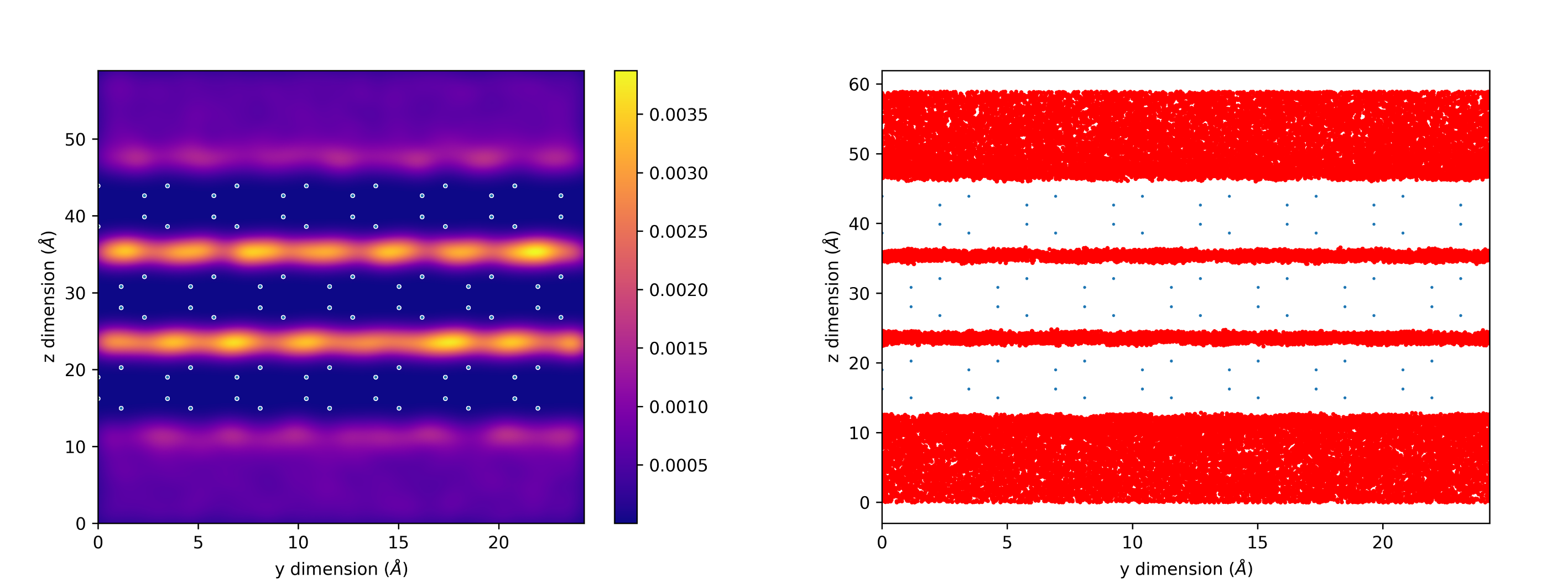

Supplement: Supplementary file 1 [file molecules-25-02526-s001.zip › supplementary material/3_5_joined.png]

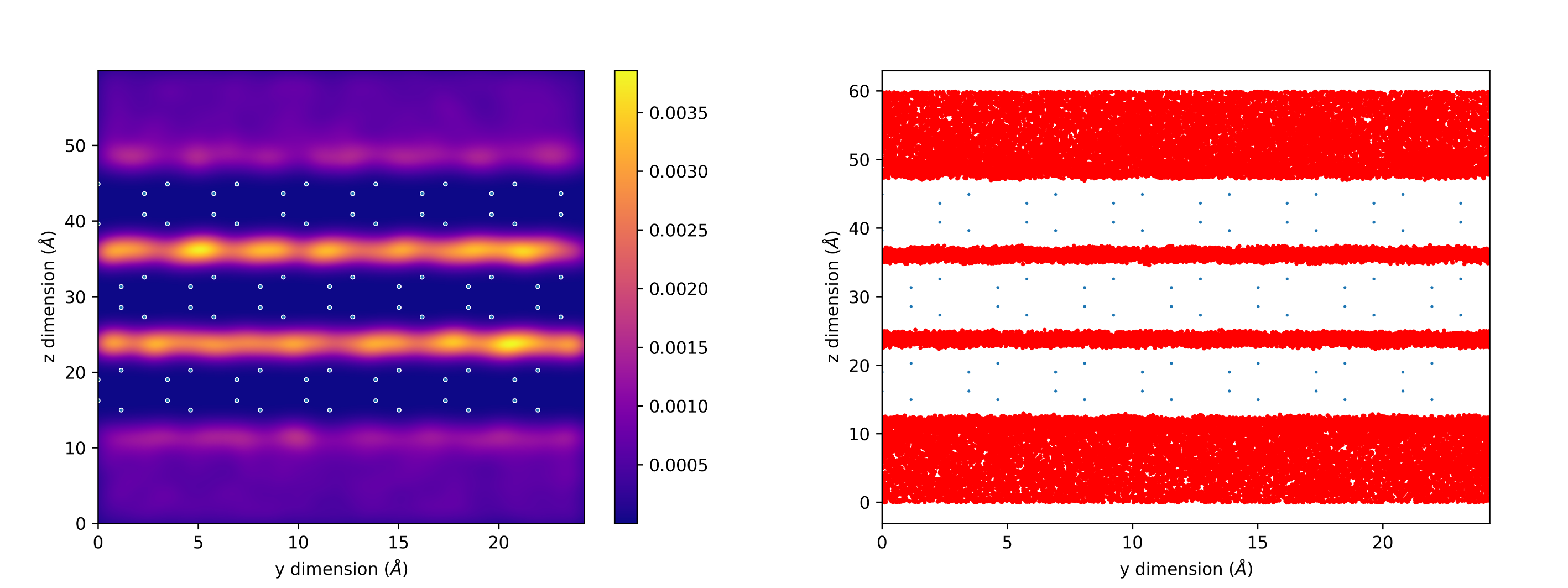

Supplement: Supplementary file 1 [file molecules-25-02526-s001.zip › supplementary material/4_0_joined.png]

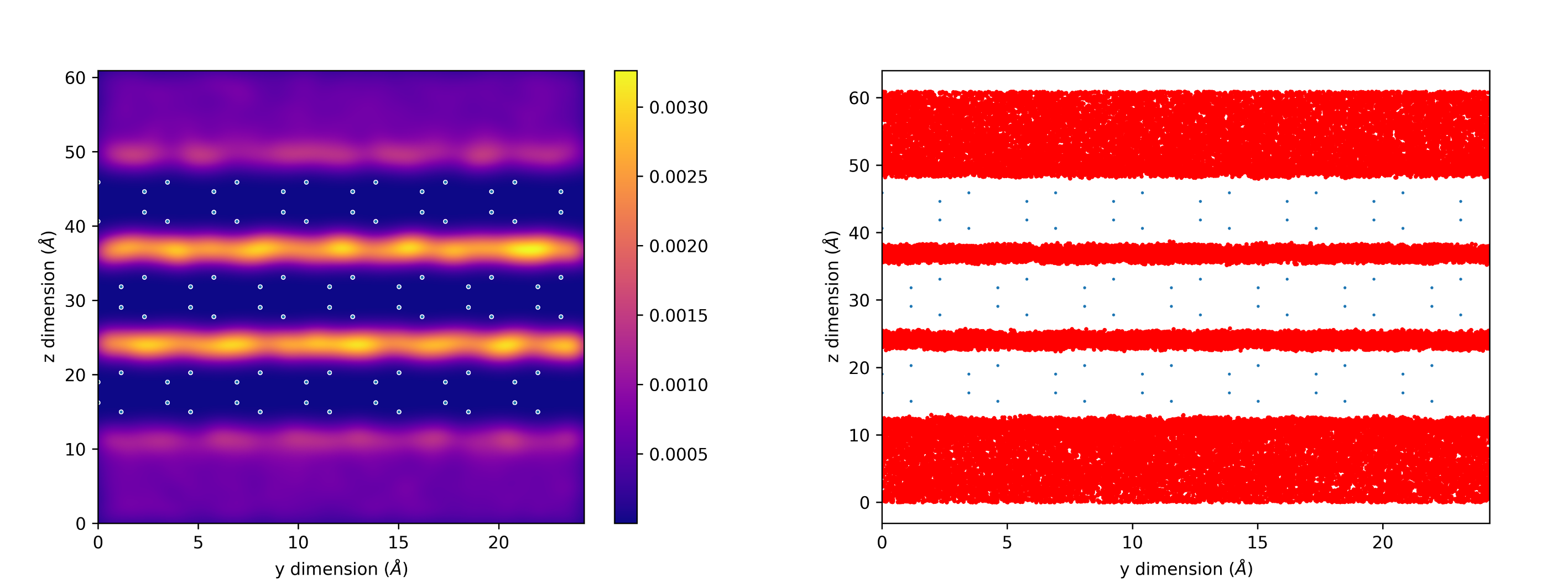

Supplement: Supplementary file 1 [file molecules-25-02526-s001.zip › supplementary material/4_5_joined.png]

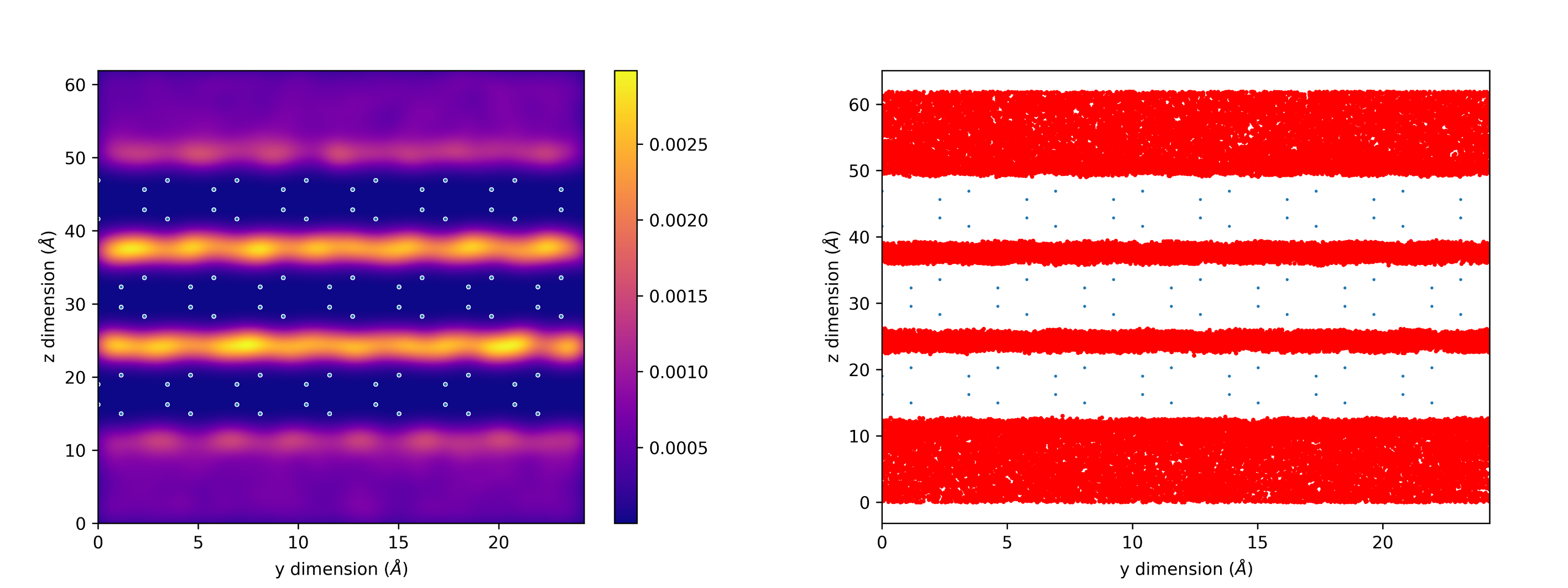

Supplement: Supplementary file 1 [file molecules-25-02526-s001.zip › supplementary material/5_0_joined.png]

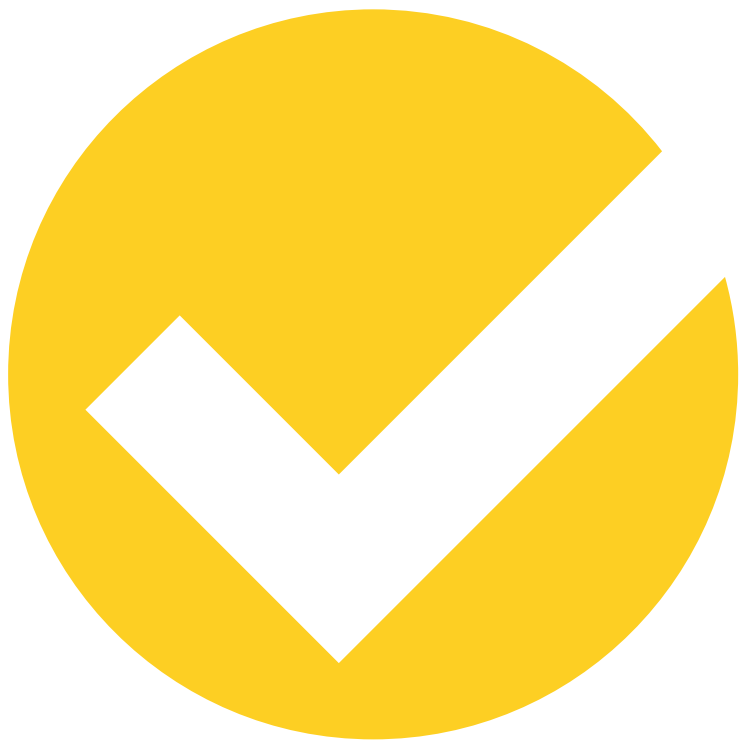

check for  
updates

Supplement: Supplementary file 1 [file molecules-25-02526-s001.zip › supplementary material/Definitions/logo-updates.pdf]

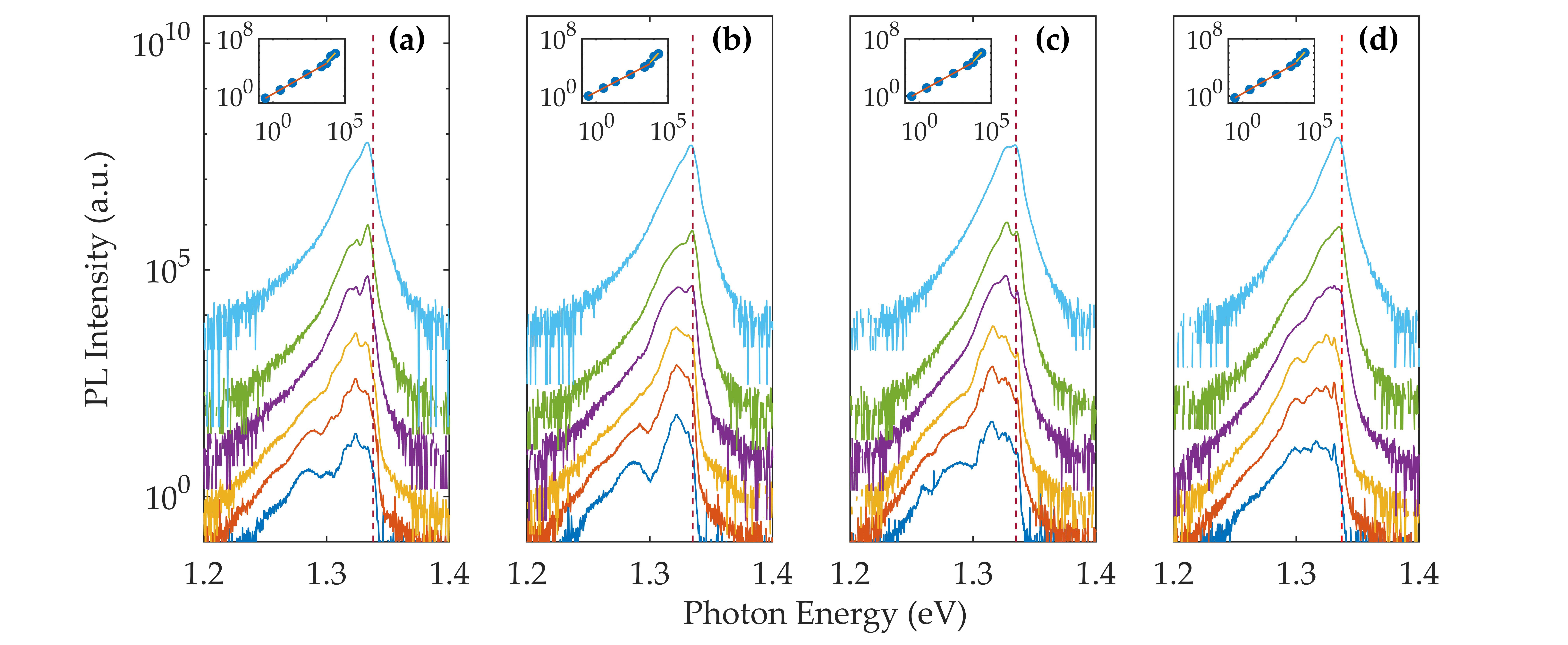

Supplement: Supplementary file 1 [file molecules-25-02526-s001.zip › supplementary material/Low_Temp_PL_Hyd.jpg]

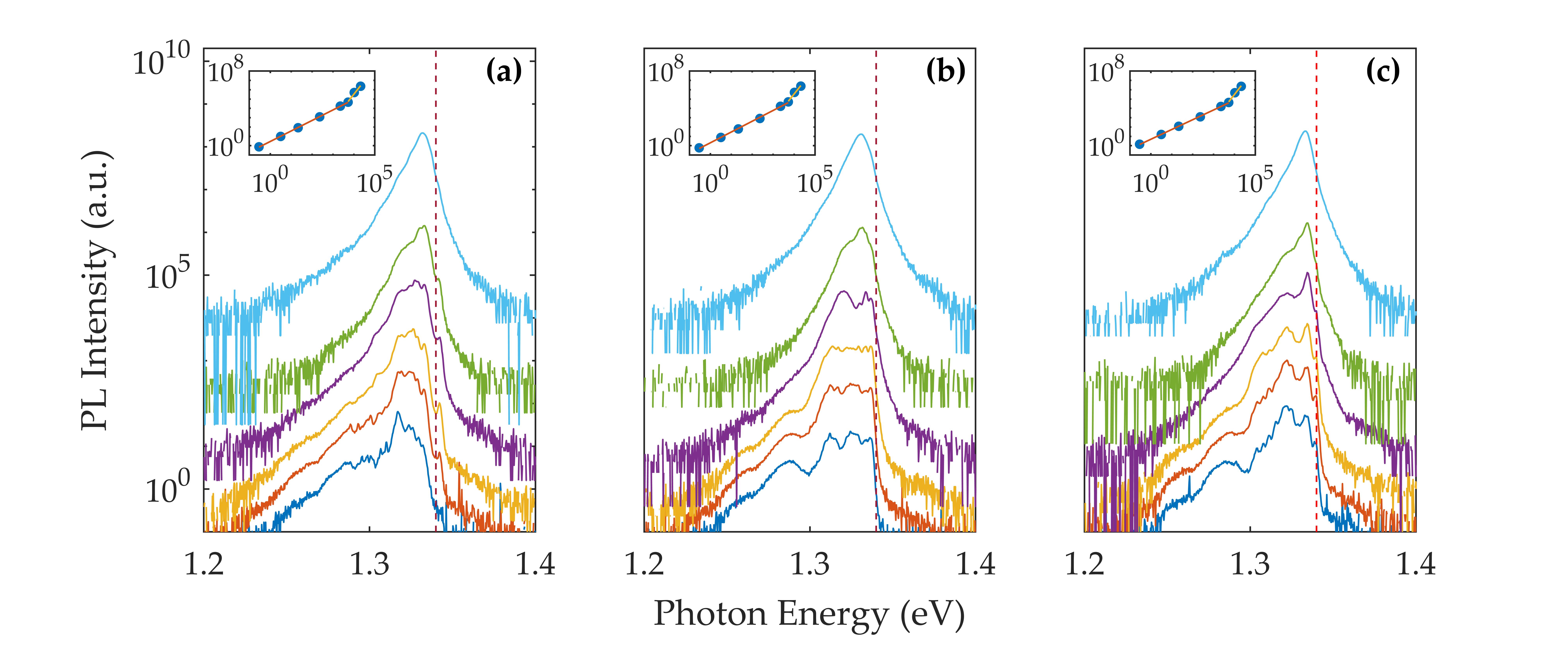

Supplement: Supplementary file 1 [file molecules-25-02526-s001.zip › supplementary material/Low_Temp_PL_Pris.jpg]

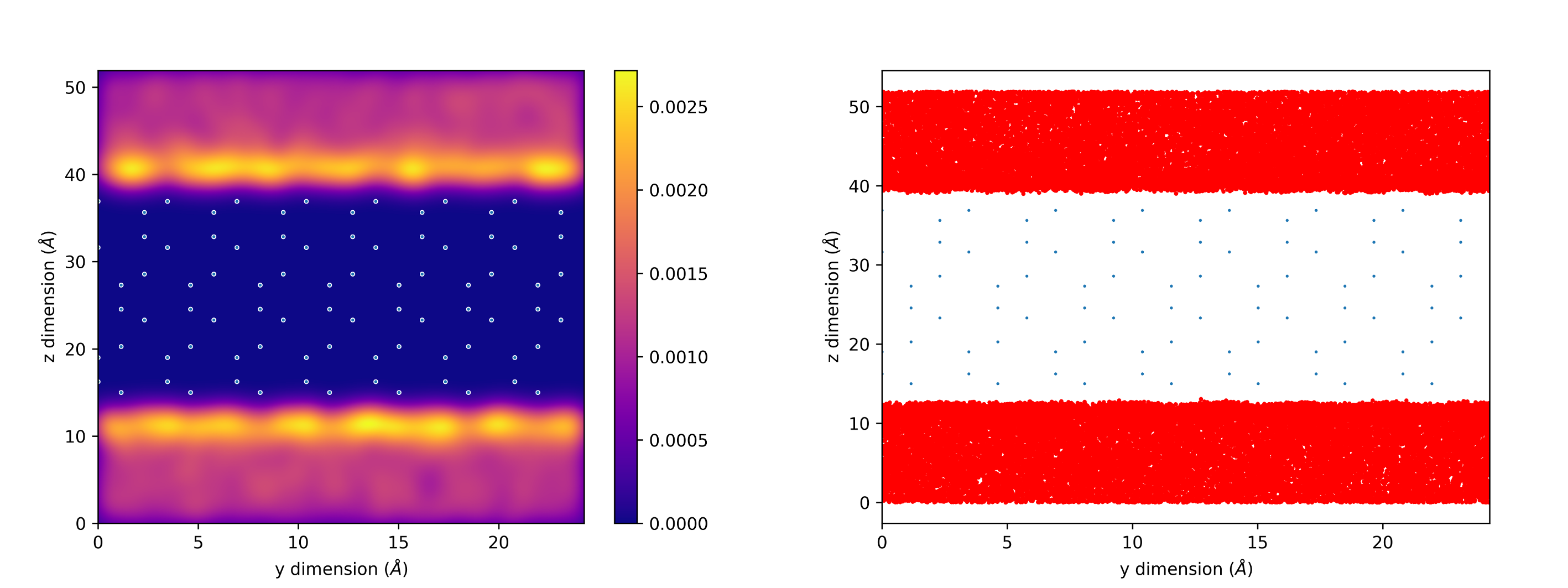

Supplement: Supplementary file 1 [file molecules-25-02526-s001.zip › supplementary material/pristine_joined.png]

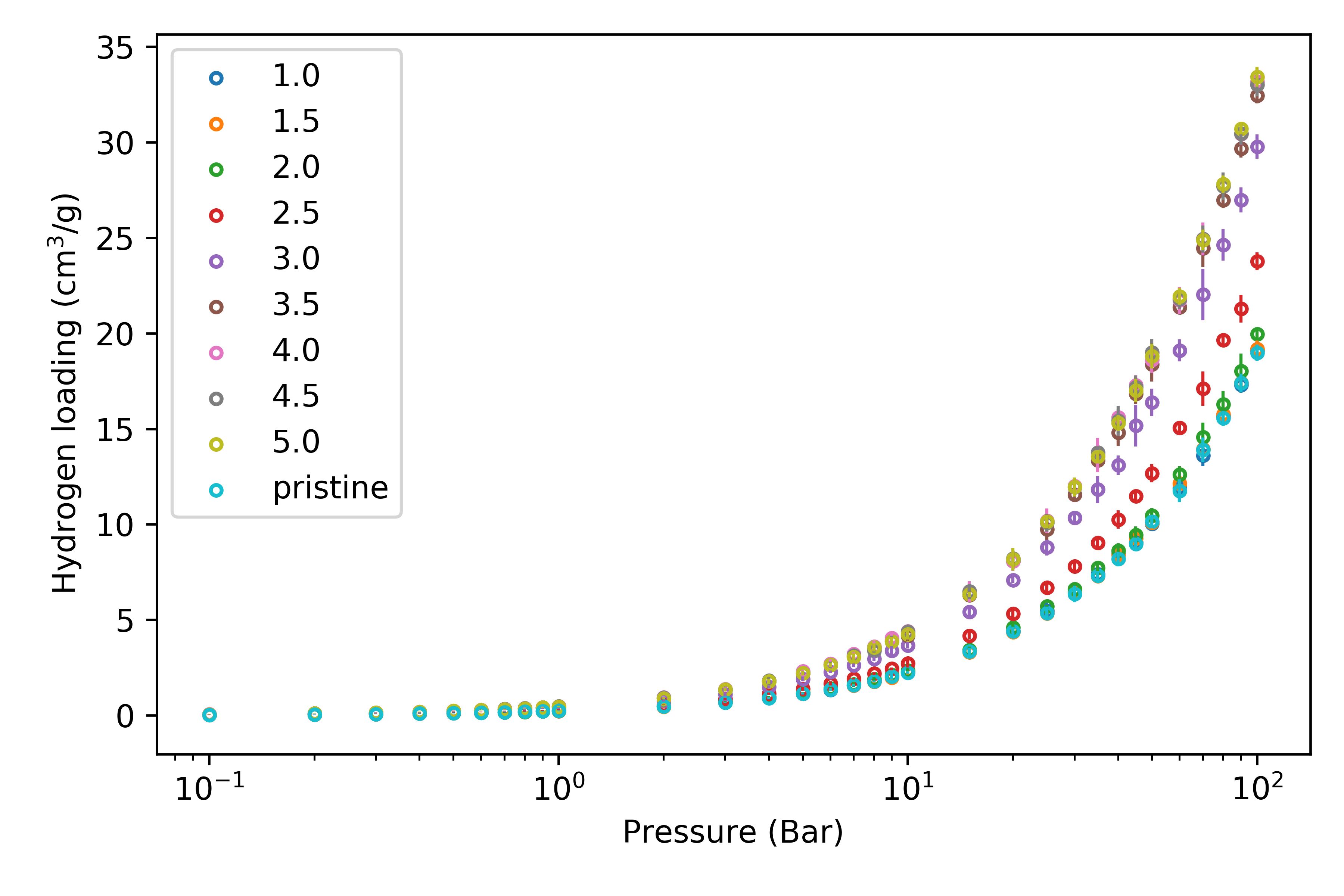

Supplement: Supplementary file 1 [file molecules-25-02526-s001.zip › supplementary material/separation_isotherm.png]

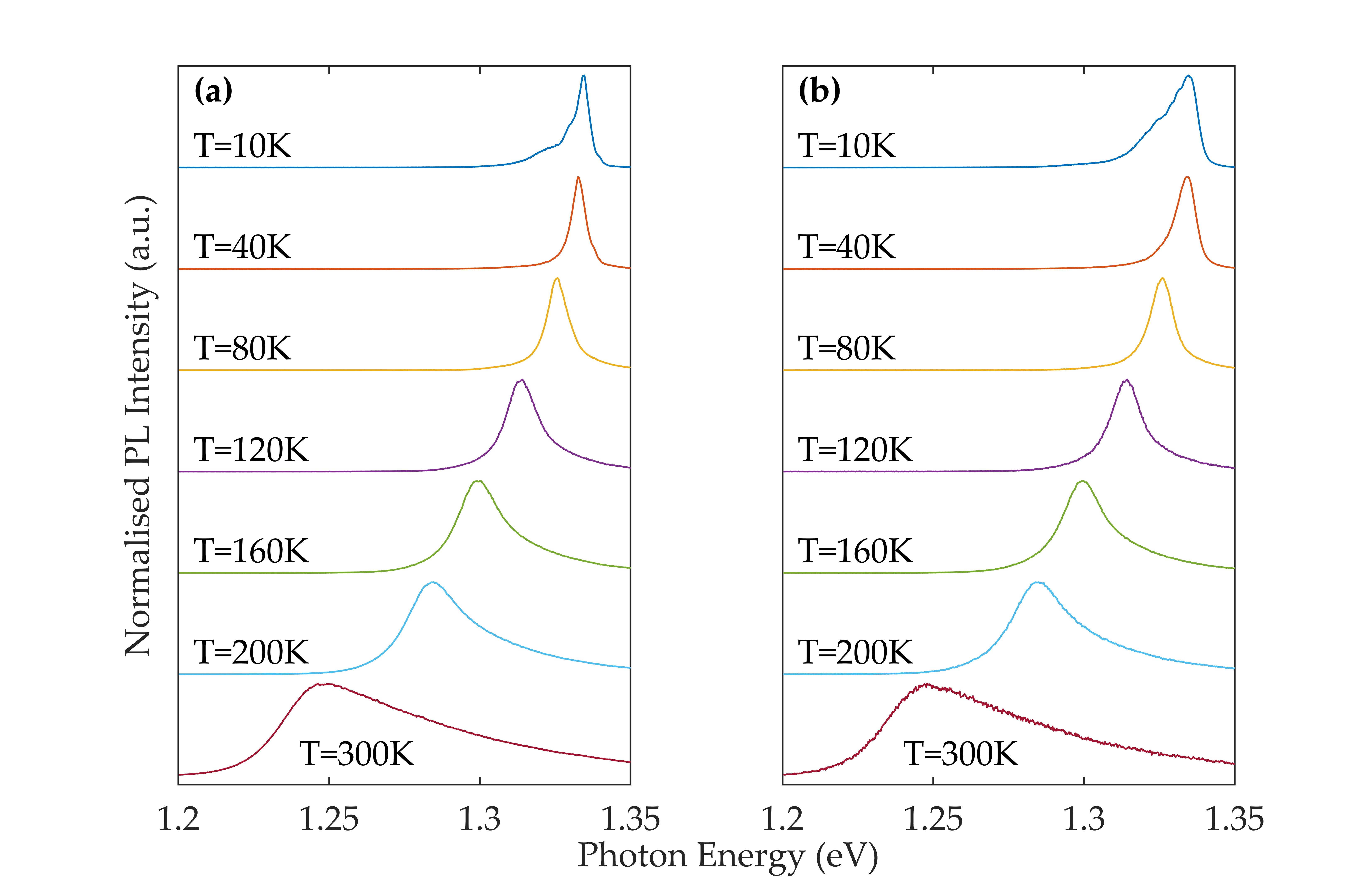

Supplement: Supplementary file 1 [file molecules-25-02526-s001.zip › supplementary material/Temp_Variation_Pris(a)_Hyd(b).jpg]
